# Early Access Provision for Innovative Medicinal Products in Oncology: Challenges and Opportunities

**DOI:** 10.3389/fonc.2020.01604

**Published:** 2020-09-02

**Authors:** Andriy Krendyukov

**Affiliations:** VP Head Global Medical Affairs, Apogenix AG, Heildelberg, Germany

**Keywords:** compassionate use, early access provision, expanded access, innovative medicinal products, oncology

An unmet medical need exists for many patients with seriously-debilitating or life-threatening diseases who cannot be treated satisfactorily by available therapeutics, particularly in oncology. Continuous efforts over the past 20 years have been made by the European Medicines Agency (EMA) and US Food and Drug Administration (FDA) to expedite the development, review and approval of Innovative Medicinal Products (InMPs) intended for treatment in oncology. For example, the FDA granted accelerated approval to 64 malignant hematology and oncology products for 93 new indications between 1992 and 2017 (1). These efforts are continuing and in recent years' programs such as the PRIority MEdicines (PRIME) scheme have been introduced by the EMA, and FDA Fast Track and Breakthrough Therapy designations in the USA. All comply with stringent risk/benefit evaluations and are intended to optimize the development of InMPs that target high unmet medical need, but even in the event of a positive competent authorities' decision, there may still be a lengthy delay before oncology patients can gain access to new therapies. Although the EU has a centralized authorization procedure, many regulations are country-specific and there are also country-level differences related to pricing and reimbursement. Furthermore, ethical considerations regarding the patient's fundamental right to have access to InMPs in a timely manner, have not yet been fully integrated into regulatory frameworks.

For oncology patients who have exhausted all treatment options, collaborative efforts between different stakeholders and regulatory agencies have led to two main options for gaining early access provision (EAP) to an InMP: participation in a clinical trial, or through a specific path known as expanded access in the USA and individual named-patient or compassionate use programs in the EU. In reality, enrolment into clinical trials can be challenging due to strict eligibility criteria and selection processes, or difficulty coping with visit schedules due to geographic location, etc. For these patients, EAP might be the only option to obtain innovative treatment in a real time oriented manner.

EAP programs are not without their challenges. Foremost among these are improving physician and patient awareness that such programs actually exist, encouraging manufacturer participation, and ensuring appropriate timing of the application. However, joint efforts between the pharmaceutical industry and international regulatory agencies have led to a number of initiatives to accelerate early patient access to certain investigational drugs (2, 3). Definitions and regulations vary by regions and by countries with the most established programs available in Europe and the USA. The EMA provides recommendations through the Committee for Medicinal Products for Human Use (CHMP) for “Compassionate Use” (2), which recognizes two situations of exceptional application of a non-licensed medicinal product to patients: those applicable for a cohort of patients as “Compassionate Use” and those for “Named Patient Use” (2) (Table 1). The early access equivalent in the USA is known as “Expanded Access,” which can be granted for three types of investigational new drug (IND) use: (1) individual patient IND use; (2) limited use for an intermediate-size IND patient population; and (3) treatment IND for widespread use (4, 7). Similarities and differences in regulatory and ethical challenges between EAP programs in Europe and the USA are illustrated in Table 1.

**Table 1:** A comparison of Early Access Provision (EAP) programs for Europe and the USA.

	**European Medicines Agency**	**US Food and Drug Administration**
Definition Compassionate use	A treatment option for patients in the European Union suffering from a disease for which no satisfactory authorized alternative therapy exists or who cannot enter a clinical trial. These programs are only put in place if the medicine is expected to help patients with life-threatening, long-lasting or seriously debilitating illnesses, which cannot be treated satisfactorily with any currently authorized medicine.	Expanded access is a potential pathway for a patient with an immediately life-threatening condition or serious disease or condition to gain access to an investigational medicinal product (drug, biologic, or medical device) for treatment outside of clinical trials when no comparable or satisfactory alternative therapy options are available.
Criteria to apply	• The medicinal product is to be made available to “*patients with a chronically or seriously debilitating disease, or a life threatening disease, and who cannot be treated satisfactorily by an authorized medicinal product*” in the European Union, • The compassionate use program is intended for a “*group of patients*,” • The medicinal product is either “*the subject of an application for a centralized marketing authorization in accordance with Article 6 of Regulation (EC)# 726/2004 or is undergoing clinical trials*” in the European Union or elsewhere.	• Patient has a serious disease or condition, or whose life is immediately threatened by their disease or condition. • There is no comparable or satisfactory alternative therapy to diagnose, monitor, or treat the disease or condition. • Patient enrollment in a clinical trial is not possible. • Potential patient benefit justifies the potential risks of treatment. • Providing the investigational medical product will not interfere with investigational trials that could support a medical product's development or marketing approval for the treatment indication.
Vs clinical trials	• Clinical trials are practically the only means of obtaining reliable and interpretable efficacy and safety data for a medicinal product. • Although safety data may be collected during compassionate use programs, such programs cannot replace clinical trials for investigational purposes. • Compassionate use is not a substitute for properly conducted trials. • Compassionate use should therefore not slow down the implementation or continuation of clinical trials intended to provide essential information relative to the benefit/risk balance of a medicinal product. • Patients should always be considered for inclusion in clinical trials before being offered compassionate use programs.	Whenever possible, an investigational medical product should be used as part of a clinical trial
Vs off-label use	Product is authorized/available and it is the physician's decision to use it outside of official indications/label.	
Vs patient support program (PSP)	PSP or disease management program is used to collect safety data of authorized products in post-approval/post-launch phase of product life cycle management.	
Different role and responsibility	Compassionate use programs are intended to facilitate the availability to patients of new treatment options under development. National compassionate use programs, making medicinal products available either on a named patient basis or to cohorts of patients, are governed by individual Member States (MS) legislation.	Sponsor to report (individual and cohort) IRB review is mandatory
Different types	• Patient cohort (compassionate use). • Individual/single patient use is different and regulated by country	• (1) Individual patient use, the most common category, in which access is granted for a single person with a serious disease and no viable alternative option and may be for emergency or non-emergency use • (2) Limited use for an intermediate size patient population not exceeding 1,000 • (3) Widespread use for a larger treatment population on the basis of a successful clinical trial result, as the drug has not yet been approved for public access
Competent authorities involved in Compassionate use/ Expanded Access Programs (4–6)	European Medicines Agency (EMA) MHRA (UK) ANSM (France) PEI and BfArM (Germany) HPRA (Ireland) Austrian Medicines and Medical Devices Agency FAMHP (Belgium) Danish Medicines Agency (Denmark) AIFA (Italy) AEMPS (Spain) MPA (Sweden) MEB (Netherlands) JAZMP (Slovenia) NOMA (Norway) Ravimiamet (Estonia) Amt für Gesundheit (Liechtenstein)	US Food and Drug Administration (US FDA)

EAP programs are intended to facilitate the availability to patients of new treatment options under development. However, uptake has been low in many countries, particularly for intermediate- to large-sized patient populations. For the period 2010–2014, a review of the three types of Expanded Access requests accepted by the FDA showed that the majority of applications were for individual patient IND (emergency), which climbed from a total of 500 in 2010, to 1,066 in 2014 (8). Individual patient IND (non-emergency) and intermediate-size IND also saw large proportionate increases, but over the 5-year period there was only one request for a Treatment IND (8). Importantly, the majority of the expanded access applications were accepted by the FDA.

A recent explorative analysis of available data from the FDA Center for Drug Evaluation and Research (CDER) and Center for Biologics Evaluation and Research (CBER) for the three expanded access categories was published by ASCO 2020 (9). The findings were compared to centralized compassionate use programs; excluding generics and biosimilars. Over the period 2014–2018, submissions for intermediate-size IND (ISIND) and treatment IND (TIND) categories were again dramatically low compared with individual patient IND (IPIND) and total applications for expanded access. There was also a decline in the number of applications from 1,886 in 2014 to 1,598 in 2018, driven largely by a reduction in the number of ISINDs. Over the same period, FDA approval of all three expanded access categories was high. The CDER approved 174 out of 183 (95%) ISINDs as well as the single TIND application, and the CBER approved 24 out of 27 (89%) ISINDs and 8 out of 10 (80%) TINDs. The trend for 2019 was similar with ISIND and TIND represented by 1.32 and 0.34%, respectively.

In Europe, Compassionate Use programs are coordinated and implemented by Member States (10). However, despite a requirement to notify the EMA about nationally-implemented programs intended for a group of patients, a CHMP Opinion on centralized Compassionate Use has only been requested six times in the past 10 years with no examples in oncology (2).

These findings indicate that applications for patient groups are very low (in general, and in comparison to individual patient use) in both Europe and the USA, thereby limiting patient access to potentially life-improving or life-saving treatments prior to InMP availability. With most applications for EAP being approved by regulatory agencies, it appears that it is the pharmaceutical companies who might be reluctant to consider early access to InMPs outside of clinical trials. A 2016 survey found that just 19% of 100 drug makers publicly posted policies about their programs for obtaining these drugs, and only one company posted information about specific procedures for making requests without listing any contact information (11).

There are a number of reasons for why it may not be possible for a manufacturer to provide a product *via* an EAP program (12). Early in their development, many compounds are not produced in large enough quantities to supply both clinical trial and EAP patients, and the former will necessarily take priority. In addition, the availability or suitability of the data may not meet the requirements of the clinical data package for early access. As EAP in most countries is provided at no cost, the financial and operational burden may limit manufacturer willingness to support such programs in oncology, and for small- and medium-sized companies it may not even be feasible. The procedure can also be time consuming. The FDA has estimated that 120 h of manpower are required for the development of an intermediate-size IND protocol (12). There are also questions concerning ethical allocation of medications in development with approval by an ethics committee obligatory for expanded access in the USA, and some countries in Europe. While all would agree that patients taking part in an EAP must provide informed, voluntary consent, many may not fully comprehend the risks involved, particularly if a neutral party like an IRB or an ethics committee is not involved. In rare cancers, the small numbers of diagnosed patients can make clinical trial recruitment difficult, and subsequently the clinical data package required for EAP consideration might be very limited or even absent.

Other potentially challenging areas manufacturers face when considering EAP in oncology relate to the data collected. One concern is that adverse events collected outside of the regulated framework of clinical trials, even in the absence of or uncertain relationships to InMP, might change the InMP safety profile and jeopardize future regulatory authority approval on clinical trial completion. In response, the FDA now only requires reporting of adverse events that occur during expanded use if there is evidence to suggest a causal relationship with the InMP (13). A paper authored by FDA officials found little evidence to support the argument that adverse events occurring as a result of expanded access treatment can jeopardize an InMP development program (14, 15).

The above concerns should be balanced by the potential advantages of making InMPs available to patients outside of a clinical trial, and the expected positive risk/benefits. EAPs can allow a manufacturer to collect clinically relevant data on InMP utilization that can be included in the review process by competent authorities. They can also generate additional safety and real-world evidence prior to launch that can help companies understand how InMP may be used in the varied population found in daily clinical practice and provide payers with the opportunity to evaluate outcomes in a real-world setting outside of clinical trials.

By capturing treatment-related adverse events prior to launch, manufacturers and regulatory agencies are better prepared to manage risk further down the line with implementation risk mitigation strategies, adapted patient screening, or other specific post-approval commitment tools, including post-approval safety studies or monitoring. For patients, the clinical benefits of EAP should outweigh any potential risk of side effects. However, drugs accessed through EAP do not undergo the scrutiny of a benefit-risk assessment provided within the regulatory framework applied to new drug submissions or clinical trial applications. Furthermore, in the absence of treatment alternatives, some oncological patients might be willing to accept higher risks in order to achieve clinical benefits, even if not yet proven in pivotal phase III clinical trials, which can have important ethical issues.

Economic aspects of EAP can affect all stakeholders: manufacturers, payers and patients with the underlying question being who should pay for access to InMPs. FDA guidance offers the ability to recover direct costs such as those of raw materials, labor, and equipment required to make the quantity of InMP required for the patient's use, but InMPs offered under an EAP cannot be priced for profit. Payers do not generally cover treatments that have not been approved by regulators. The full production and supply costs of EAP are therefore currently borne by the manufacturer and/or patient. Given that EAP may not interfere with or replace clinical trials, i.e., patients should always be considered for inclusion into a clinical trial first, they are not an alternative to costly clinical trials (16, 17). However, the unreimbursed time and resources involved in treating patients through expanded access can potentially be offset by a number of opportunities including the possibility of earlier conversations with formulary committees, and the ability to capture valuable data particularly in patient subtypes not included in clinical trials, potentially leading to broader indications.

Some EU Member States have nationalized programs in place that allow companies to access markets through the donation of medication for at-risk groups of patients, such as the German and Italian Compassionate Use Programs and the UK Early Access to Medicines Scheme, although these initiatives carry no revenue for the donating organization. Other schemes, such as the French Temporary Utilization Program (Autorisation Temporaire d'Utilisation, ATU), allow the pharmaceutical industry to access a market pre-authorization and derive revenue from a product, albeit for a short period of time (18, 19). Canada operates a program similar to EAP that is known as Special Access Program (SAP). This considers requests from practitioners for access to unauthorized InMP for treatment, diagnosis, or prevention of serious or life-threatening conditions when conventional therapies have been considered and ruled out, have failed, are unsuitable or unavailable. Like other EAP, products accessed through SAP do not undergo the scrutiny of a benefit-risk assessment provided within the regulatory framework applied to InMP submissions or clinical trial applications.

To use EAP effectively, pharmaceutical companies should collaborate closely with a range of stakeholders early in InMP development (from Phase 2 trials onwards) to define inclusion criteria for compassionate use. This is particularly important in oncology as the InMP mechanism of action may extend to more than one type of cancer and thus to EAP requests from patients suffering from a different form of cancer to that being evaluated in the registration trials. Outcome measurements should also be decided early on so that they can be used to support accelerated regulatory approval and economic assessments. In the USA, the 21st Century Cures Act, which came into effect in 2017, now requires expanded access policies to be made public after Phase 2 of clinical testing begins. Although currently few in number, there are examples where expanded access data have been used to support approval of InMPs. For example, the submitted data package for the autologous gene therapy for adenosine deaminase deficiency (autologous CD34+ enriched cell fraction) included efficacy and safety data from 18 patients, three of whom received the product as part of a compassionate use program (20); the drug was subsequently approved by the EMA in 2016. There is now increasing support from patient advocacy groups (21) and the FDA (22) for data collected in EAP settings to be considered as part of the evidence base for safety and efficacy when clinical trials are impossible, or as an adjunct to data from traditional clinical research trials.

There is considerable variation between countries in the need for review of expanded use by research ethics committees. Those in favor of mandatory ethical review state that compassionate use is different from standard clinical care and can involve significant research aspects, as well as being based on unapproved drugs with unproven safety and efficacy (23). The issue of EAP funding also raises ethical concerns related to equal access, in that some patients will be able to raise the required funds whereas others will not. This issue must be addressed if such programs are to become more widespread (24).

In Europe, a common approach to EAP is required, which must be centralized to ensure consistent and fair access for all patients. A recent pilot independent Compassionate Use Advisory Committee set up for an investigational agent in oncology suggests that this might be best served by a multidisciplinary group including independent medical experts, bioethicists, and patient representatives (24). For timely response to EAP requests, companies should plan to implement a comprehensive strategy at a global level (25). Practicing oncologists and oncology pharmacists may not be very familiar with EAP policies or the procedures involved, yet are often the first point of contact for a patient seeking access. Consideration for education, training, and availability of easy-to-use on-line tools should therefore form an integral part of the EAP road-map, and be expanded to the broader range of stakeholders including key medical societies and patient advocacy groups. This will include information on how to lodge a request, clear roles and responsibilities charter between treating physician, responsible pharmacist, competent authorities and manufacturer, as well as specific details on how to collect, evaluate and report adverse events or other clinical data. The pharmaceutical industry and regulatory agencies might consider close collaboration with regards to clinical data collection from EAPs as supporting evidence to be included and considered during the pre-authorization review process.

Implementation of an EAP is a strategic decision and pharmaceutical companies should consider the implications for each stage of InMP clinical development and where possible, incorporate EAP into drug development plans so that companies are ready to meet the needs of patients not eligible for enrolment in clinical trials. When carefully planned the benefits for patients, manufacturers and payers can outweigh any risks.

## Conclusion

EAPs can support oncology patients with access to InMP at the same time as providing valuable evidence, complementary to clinical trials. However, despite high approval rates by regulatory agencies, applications for group EAP remain low and represent an underutilized opportunity for oncological patients to obtain early access to care. Educational effort is required to inform all stakeholders of the value of these programs and the regulatory framework to follow.

## Author Contributions

The author confirms being the sole contributor of this work and has approved it for publication.

## Conflict of Interest

AK was employed by the company Apogenix AG and declares that the research was conducted in the absence of any commercial or financial relationships that could be construed as a potential conflict of interest.
